# The psychological impact of COVID-19 pandemic on medical staff in Guangdong, China: a cross-sectional study

**DOI:** 10.1017/S0033291720002561

**Published:** 2020-07-06

**Authors:** Huajun Wang, Daozheng Huang, Huigen Huang, Jihui Zhang, Lan Guo, Yuting Liu, Huan Ma, Qingshan Geng

**Affiliations:** 1 Intensive Care Unit, Guangdong Provincial People's Hospital, Guangdong Academy of Medical Sciences, Guangzhou, Guangdong, China; 2 Department of Critical Care Medicine, Guangdong Provincial People's Hospital, Guangdong Academy of Medical Sciences, Guangdong Provincial Geriatrics Institute, Guangzhou, Guangdong, China; 3 Guangdong Provincial People's Hospital, Guangdong Academy of Medical Sciences, Guangzhou, Guangdong, China; 4 Department of Psychiatry, Faculty of Medicine, The Chinese University of Hong Kong, Shatin, NT, Hong Kong SAR, China; 5 Guangdong Provincial Key Laboratory of Coronary Heart Disease Prevention, Guangdong Cardiovascular Institute, Guangdong Provincial People's Hospital, Guangdong Academy of Medical Sciences, Guangzhou, Guangdong, China

**Keywords:** Anxiety, coronavirus disease 2019, COVID-19, medical staff, psychological status

## Abstract

**Background:**

During previous pandemic outbreaks, medical staff have reported high levels of psychological distress. The aim of the current study was to report a snapshot of the psychological impact of the coronavirus disease 2019 (COVID-19) pandemic and its correlated factors on medical staff in Guangdong, China.

**Methods:**

On the 2nd and 3rd February 2020, soon after the start of the COVID-19 pandemic, we surveyed medical staff at four hospitals in Guangdong, China, to collect demographic characteristics, Hospital Anxiety and Depression Scale (HADS), Perceived Stress Scale (PSS-14), and Insomnia Severity Index (ISI) scores.

**Results:**

Complete responses were received from 1045 medical staff. Respondents were divided into high- and low-risk groups according to their working environment of contacting with potential or confirmed COVID-19 cases. The proportion of staff with anxiety (55.4% *v.* 43.0%, *p* < 0.001) or depression (43.6% *v.* 36.8%, *p* = 0.028) was significantly higher in the high-risk group than the low-risk group. The percentage of staff with severe anxiety was similar in the two groups. Doctors were more susceptible to moderate-to-severe depressive symptoms. The high-risk group had higher levels of clinical insomnia (13.5% *v*. 8.5%, *p* = 0.011) and were more likely to be in the upper quartile for stress symptoms (24.7% *v*. 19.3%, *p* = 0.037) than the low-risk group. Additionally, work experience negatively correlated with insomnia symptoms.

**Conclusions:**

It is important for hospitals and authorities to protect both the physical and psychological health of medical staff during times of pandemic, even those with a low exposure risk.

## Introduction

In late December 2019, clusters of pneumonia cases with unknown cause emerged in Wuhan, China. Sequencing of lower respiratory lavage fluid indicated that the disease was caused by a novel coronavirus in humans that was subsequently named 2019 novel coronavirus (2019-nCoV) by the World Health Organization (WHO, 2020a). The novel coronavirus is thought to be a relative of the deadly severe acute respiratory syndrome (SARS) and Middle East respiratory syndrome viruses. These coronaviruses are all characterised by flu-like symptoms, including fever, cough, and chills – they can also cause severe respiratory illness and death (WHO, 2020a). During Spring Festival in China, hundreds of thousands of people left Wuhan, often via public transport, potentially carrying the virus with them. As a result, the virus is now rapidly spreading worldwide.

On the 31st January 2020, the WHO declared the Coronavirus Disease 2019 (COVID-19) outbreak in China constituted a Public Health Emergency of International Concern (PHEIC) (WHO, 2020b). By the 1st February 2020, 14 380 cases of COVID-19 were confirmed nationwide by the Chinese government (including medical staff), and another 19 544 cases were suspected (National Health Commission, 2020). Shortly after, hundreds of cases were confirmed in Thailand, Japan, South Korea, German, and the United States of America (WHO, 2020c).

Because of the urgency of the situation, most current research has mainly focused on the clinical manifestations and epidemiology of COVID-19. The psychological impact and distress associated with COVID-19 is largely neglected. Dealing with a highly infectious disease outbreak entails a risk of infection, psychological stress, and emotional challenges for all those involved. A study of the 2003 SARS outbreak showed medical staff in Toronto, Canada, suffered from high levels of psychological stress while treating patients (Maunder, 2004). About 10% of medical staff in Beijing have experienced high levels of post-traumatic stress symptoms since the SARS outbreak (Wu et al., 2009). High burdens of stress among medical staff were also documented during the Ebola outbreak in 2014 (Lehmann et al., 2016). Furthermore, during the H1N1 influenza pandemic in Japan, medical staff in high-risk work environments felt more anxious and more exhausted than those in lower risk environments (Matsuishi et al., 2012). Other studies have also shown an association between negative life-events (including occupational stress) and higher levels of anxiety and psychological distress (Shigemura, Tanigawa, & Nomura, 2012; Weinberg & Creed, 2000).

To date, there are few studies reporting on the psychological impact of COVID-19 in a large population. Lai and colleagues have found a considerable proportion of medical staff who worked around patients with COVID-19 in several cities around China to have symptoms of depression, anxiety, insomnia, and distress (Lai et al., 2020). Medical staff in high risk departments are potentially more susceptible to psychological disorders (Lu, Wang, Lin, & Li, 2020), and compared with many other occupations, a high proportion of medical staff report of poor sleep quality (Huang & Zhao, 2020). Sleep problems and stress are closely related to the development of depression and anxiety (Cox & Olatunji, 2020; Ford & Kamerow, 1989; Kendler, Thornton, & Gardner, 2000).

Medical staff nearly always receive intensive infection control training before dealing with highly infectious patients; however, their safety can never be guaranteed. In addition to their fears of personal contamination, medical staff may have to cope with other stresses, including the deaths of colleagues, threats to their family's safety, and working excessive hours. Some may also suffer from stigmatisation and violence targeted against health professionals (Lancet, 2020).

Given the possibility of future pandemics, the psychological impact of the current COVID-19 pandemic on medical staff should be evaluated. This study reports the immediate stress and psychological impact of COVID-19 on medical staff in several seriously affected hospitals in Guangdong province, China, around 1 month after the first Chinese case was reported. The number of confirmed cases in Guangdong province has increased to 604 as of 2nd February 2020, with the second rank in China (Health Commission, 2020).

## Methods

### Study sample

Medical staff (doctors, nurses, and auxiliary staff) in Guangdong Provincial People's Hospital, Luoding People's Hospital, Yingde People's Hospital, and Huizhou Sixth People's Hospital, all in Guangdong province, completed electronic questionnaires between the 2nd and 3rd February 2020. The questionnaire was distributed to all staff who worked in the fever clinic, emergency department, intensive care unit (ICU), infectious disease department, and three to four medical wards or auxiliary departments by administrators (e.g. department head nurse) at each hospital via WeChat™ (Tencent Inc., Shenzhen) work groups.

Participation was strictly voluntary and all responses were anonymous. The response rate for the questionnaire was calculated according to the total number of individuals in each WeChat™ group.

### Content of questionnaire

A cover note, stating that the purpose of the survey was to examine the mental health status of the medical staff during the COVID-19 pandemic, was distributed with the questionnaire. The note also stated that the results of the survey would be published, and that the respondent's answers would remain anonymous. The survey consisted of questions on demographic characteristics and psychological status.

The surveyed demographic characteristics included gender, education, job, years of employment in the current role, working department, and work environment during the COVID-19 pandemic. Jobs were categorised as either medical doctor, nurse, or auxiliary staff (radiological technologists, clinical laboratory technicians, pharmacists, dieticians, physical therapists, office workers, or clinical clerks). Work environment was categorised as high-risk (direct contact with a confirmed or suspected case of COVID-19) or low-risk (no direct contact).

To assess the staff's psychological distress, the Hospital Anxiety and Depression Scale (HADS), Perceived Stress Scale (PSS-14), and Insomnia Severity Index (ISI) were used.

The HADS was devised 30 years ago by Zigmond and Snaith (1983) to measure anxiety and depression in a general medical population (see online Additional File 1). Many studies have confirmed the validity of the HADS in the setting for which it was designed. The HADS consists of 14 items, divided in two seven-item subscales measuring anxiety (HADS-A) and depression (HADS-D). Items are scored on a 4-point Likert scale ranging from 0 to 3, providing total subscale scores from 0 to 21. A score ⩽7 corresponds to ‘no depression or anxiety’, a score of 8–10 is ‘minor depression/anxiety’, a score of 11–15 is ‘moderate depression/anxiety’, and a score ⩾16 is defined as ‘severe depression/anxiety’.

Perceived stress status was evaluated with the Chinese version of PSS-14 (see online Additional File 2) (Cohen, Kamarck, & Mermelstein, 1983), seven positive and seven negative, which has been validated in Chinese cardiac patients (Leung, Lam, & Chan, 2010). The PSS was developed to appraise whether respondents considered their life to be unpredictable, uncontrollable, or overloaded. Each item was scored on a 5-point Likert scale ranging from 0 = never to 4 = very often. PSS scores were obtained by reversing the scores on the seven positive items (e.g. 0 = 4, 1 = 3, 2 = 2, 3 = 1, and 4 = 0) (items 4, 5, 6, 7, 9, 10, and 13), and then summing across all scale items. A higher score represents a higher stress level and also an increased likelihood that environmental demands exceed an individual's ability to cope. As there is a lack of studies proposing a standard cut-off score to diagnose or grade stress, we categorised the PSS scores into four quartiles. The lower quartile includes scores <20, the second quartile ranges from 20 to 26, the third quartile ranges from 27 to 29, and the upper quartile includes scores that are >29 out of a possible 56.

The ISI is a commonly used seven-item psychometrically validated measure used to perceive the severity of insomnia symptoms and associated functional impairment. A 5-point Likert scale (0 = not at all, 4 = extremely) is used to rate each of these items, yielding to a total score ranging from 0 to 28, with scores of 0–7 indicating absence of insomnia, 8–14 indicating sub-threshold insomnia symptoms, 15–21 indicating moderate insomnia, and 22–28 indicating severe insomnia (Morin, Beaulieu-Bonneau, LeBlanc, & Savard, 2005). The Chinese version of the ISI questionnaire was used in this study (see online Additional File 3). This version has good internal consistency, test–re-test reliability, and convergent validity in Chinese population (Chung, Kan, & Yeung, 2011; Wong et al., 2017).

### Statistical analysis

All analyses were performed using SPSS (version 11.0, SPSS Inc., Chicago). Descriptive statistics for the HADS, PSS, and ISI scores are presented as medians and interquartile ranges. The HADS, PSS, and ISI scores were converted from continuous variables to dichotomous variables based on their cut-offs as mentioned above: HADS divided as ‘no-minor anxiety/depression (HADS ⩽ 10)’ and ‘moderate-severe anxiety/depression (10 < HADS)’, PSS divided as ‘1–3 quartile (PSS ⩽ 29)’ and ‘highest quartile (29 < PSS)’ and ISI score divided as ‘no-subthreshold insomnia (ISI ⩽ 14)’ and ‘clinical insomnia (14 < ISI)’. Normality was tested by the Shapiro–Wilk test. Because HADS, PSS, and ISI scores tend to have skewed distributions, we used Mann–Whitney *U* test to assess differences in HADS, PSS, and ISI scores by work environment. Categorical variables were expressed as percentages and analysed using Pearson's χ^2^ test. Univariate comparisons were conducted by univariate logistic regression to assess variables associated with the degree of anxiety and depression, stress status, or severity of insomnia. Multivariate logistic regression analysis was employed to assess the factors most closely associated with staff's degree of anxiety and depression severity, stress status, and sleep disturbances. We treated groupings based on HADS, PSS, and ISI scores as dependent variables. Demographics and variables significantly associated with dependent variables in univariate analyses were treated as independent variables. Spearman correlation analysis was used to identify correlations among HADS, PSS and ISI in all subjects. All statistical tests were two-sided and *p* < 0.05 was considered significant.

## Results

The questionnaire received a total of 1049 responses. The response rate was 80.1% from the fever clinic, emergency department, ICU, and infectious disease departments, and 70.3% from the wards/auxiliary departments. Of the responses, four were excluded due to ⩾1 missing answers, leaving 1045 questionnaires (99.6%) for analysis.

### Sample characteristics

Table 1 presents the respondents' demographic and professional characteristics, along with their scores and classifications on psychological status measures. Most respondents were female (85.8%) with an education level of ‘college’ (56.4%). About 74% of respondents were nursing staff. At most, 30.9% of respondents worked in the front-line departments (such as the fever clinic, emergency department, ICU, or infectious disease department). However, 401/1045 respondents (38.4%) said that they had been in direct contact with a patient confirmed, or suspected to have, COVID-19. This indicates that there are many staff in addition to those listed above departments, who are at risk during a pandemic.

**Table 1.** Sample characteristics and psychological status (*N* = 1045)

Demographics and psychological status	
Gender	
Male	148 (14.2)
Female	897 (85.8)
Education	
Junior college	411 (39.3)
College	589 (56.4)
Master and above	45 (4.3)
Job	
Doctor	149 (14.3)
Nurse	773 (74.0)
Others	123 (11.7)
Years of working	
<1 year	81 (7.8)
2–3 years	160 (15.3)
4–5 years	121 (11.6)
>5 years	683 (65.3)
Department (multiple choices)	
Fever clinic	76 (7.3)
Emergency	123 (11.8)
ICU	45 (4.3)
Infections department	78 (7.5)
Others	772 (73.9)
Work environment	
High risk	401 (38.4)
Low risk	644 (61.6)
Psychological distress	
HADS-A score	7 (5–10)
No-minor anxiety (HADS-A ⩽ 10)	836 (80.0)
Moderate-severe anxiety (HADS-A > 10)	209 (20.0)
HADS-D score	6 (3–9)
No-minor depression (HADS-D ⩽ 10)	903 (86.4)
Moderate-severe depression (HADS-D > 10)	142 (13.6)
PSS score	26 (19.5–29)
ISI score	7 (5–12)
No-subthreshold insomnia (ISI ⩽ 14)	936 (89.6)
Clinical insomnia (ISI > 14)	109 (10.4)

ICU, intensive care unit; HADS-A, Hospital Anxiety and Depression Scale-Anxiety; HADS-D, Hospital Anxiety and Depression Scale-Depression; PSS, Perceived Stress Scale; ISI, Insomnia Severity Index.

Values are given as *N* (%), continuous variables as score are given as medians and interquartile ranges; jobs classified as medical doctor, nurse, or auxiliary staff (pharmacists, dieticians, physical therapists, office workers, or clinical clerks).

### Factors associated with medical staff's anxiety and depression severity

Of the 499 (47.8%) respondents who presented HADS-A scores >7, 209 (20.0% of the entire sample) presented scores >10, indicative of moderate to severe anxiety. Among all respondents, 412 (39.4%) presented with depression and 142 (13.6%) with moderate to severe depression. Higher HADS scores were found among staff in the high-risk grouping, indicating a higher average level of anxiety and depression (HADS-A: 8 [5–10] *v.* 7 [4–9], *p* < 0.001; HADS-D: 7 [4–9] *v.* 6 [3–9], *p* = 0.001). A higher proportion of staff had anxiety or depression in the high-risk group than in the low-risk group (55.4% *v.* 43.0%, *p* < 0.001; 43.6% *v.* 36.8%, *p* = 0.028), while the percentage with severe anxiety was similar (3.0% *v.* 1.6%, *p* = 0.115).

Univariate analyses (Tables 2 and 3) revealed a few variables to be statistically associated with the medical staff's anxiety and depression severity. Subsequent binary multivariate logistic regression analysis showed the odds of being assessed with moderate or severe anxiety (10 < HADS-A) (rather than no anxiety or minor anxiety, HADS-A ⩽ 10) was 1.59 times [95% confidence interval (CI) 1.17–2.16, *p* = 0.003]; and the odds of being assessed with moderate or severe depression (10 < HADS-D) (rather than no depression or minor depression, HADS-D ⩽ 10) was 1.48 times (95% CI 1.03–2.11, *p* = 0.033) higher in the high-risk group compared to the low risk group, after adjustment. The odds of being assessed with moderate or severe depression (rather than no-minor depression) was 2.15 times (95% CI 1.02–4.56, *p* = 0.045) greater among doctors compared to any other position; however, this difference was insignificant after adjustment (odds 2.11; 95% CI 0.96–4.64, *p* = 0.065).

**Table 2.** Variables associated with the hospital staff's anxiety severity (*N* = 1045)

Independent variables	HADS-A ⩽ 10^a^（*N* = 836）	HADS-A > 10 （*N* = 209）	Univariate analyses	
			Crude OR (95% CI)^b^	*p*-value
Gender, *n* (%)				
Male	125 (84.5)	23 (15.5)	0.15 (0.44–1.13）	0.15
Female	711 (79.3)	186 (20.7)	–	–
Education level, *n* (%)				
Junior college	321 (78.1)	90 (21.9)	–	–
College	477 (81.0)	112 (19.0)	0.84 (0.61–1.14)	0.26
Master and above	38 (84.4)	7 (15.6)	0.66 (0.28–1.52)	0.33
Job, *n* (%)				
Doctor	119 (79.9)	30 (20.1)	1.38 (0.73–2.60)	0.32
Nurse	613 (79.3)	160 (20.7)	1.43 (0.85–2.40)	0.18
Others	104 (84.6)	19 (15.4)	–	–
Years of working, *n* (%)				
<1 year	70 (86.4)	11 (13.6)	0.63 (0.33–1.23)	0.18
2–3 years	127 (79.4)	33 (20.6)	1.05 (0.68–1.60)	0.84
4–5 years	92 (76.0)	29 (24.0)	1.27 (0.80–2.00)	0.31
>5 years	547 (80.1)	136 (19.9)	–	–
Department (multiple choices), *n* (%)				
Fever clinic	56 (73.7)	20 (26.3)	1.47 (0.86–2.52)	0.16
Emergency	100 (81.3)	23 (18.7)	0.91 (0.56–1.47)	0.70
ICU	36 (80.0)	9 (20.0)	1.00 (0.47–2.11)	1.00
Infections department	65 (83.3)	13 (16.7)	0.79 (0.43–1.46)	0.45
Others	619 (80.2)	153 (19.8)	0.96 (0.68–1.35)	0.81
Work environment (high risk), *n* (%)				
High risk	302 (75.3)	99 (24.7)	1.59 (1.17–2.16)	0.003
Low risk	534 (82.9)	110 (17.1)	–	–

HADS-A, Hospital Anxiety and Depression Scale-Anxiety; ICU, intensive care unit.

a As reference.

b Odds ratios (95% CI).

**Table 3.** Variables associated with the hospital staff's depression severity (*N* = 1045)

Independent variables	HADS-D ⩽ 10^a^ (*N* = 903)	HADS-D > 10 (*N* = 142)	Univariate analyses		Logistic regression analysis^b^	
			Crude OR (95% CI)^c^	*p*-value	Adjusted OR (95% CI)^c^	*p*-value
Gender, *n* (%)						
Male	125 (84.5)	23 (15.5)	1.20 (0.74–1.95)	0.46	1.07 (0.57–2.01)	0.84
Female	778 (86.7)	119 (13.3)	–	–	–	–
Education level, *n* (%)						
Junior college	353 (85.9)	58 (14.1)	–	–		
College	511 (86.8)	78 (13.2)	0.93 (0.64–1.34)	0.69		
Master and above	39 (86.7)	6 (13.3)	0.94 (0.38–2.31)	0.89		
Job, *n* (%)						
Doctor	123 (82.6)	26 (17.4)	2.15 (1.02–4.56)	0.05	2.11 (0.96–4.64)	0.07
Nurse	668 (86.4)	105 (13.6)	1.60 (0.83–3.07)	0.16	1.66 (0.85–3.24)	0.14
Others	112 (91.1)	11 (8.9)	–	–	–	–
Years of working, *n* (%)						
<1 year	71 (87.7)	10 (12.3)	0.84 (0.42–1.69)	0.63		
2–3 years	144 (90.0)	16 (10.0)	0.66 (0.60–1.13)	0.15		
4–5 years	103 (85.1)	18 (14.9)	1.04 (0.61–1.80)	0.88		
>5 years	585 (85.7)	98 (14.3)	–	–		
Department (multiple choices), *n* (%)						
Fever clinic	62 (81.6)	14 (18.4)	1.48 (0.82–2.73)	0.20		
Emergency	101 (82.1)	22 (17.9)	1.46 (0.88–2.40)	0.14		
ICU	38 (84.4)	7 (15.6)	1.18 (0.52–2.70)	0.69		
Infections department	66 (84.6)	12 (15.4)	1.17 (0.62–2.23)	0.63		
Others	676 (87.6)	96 (12.4)	0.70 (0.48–1.03)	0.07		
Work environment (high risk), *n* (%)						
High risk	335 (83.5)	66 (16.5)	1.47 (1.03–2.10)	0.033	1.48 (1.03–2.11)	0.033
Low risk	568 (88.2)	76 (11.8)	–	–	–	–

HADS-D, Hospital Anxiety and Depression Scale-Depression; ICU, intensive care unit.

a As reference.

b Multivariate logistic regression analysis with dependent variable and independent variables ‘gender and the statistically significant variables of the univariate comparisons’.

c Odds ratios (95% CI).

### Factors associated with medical staff's perceived level of stress

Binary logistic regression analysis revealed that work environment variables were significantly and independently associated with medical staff's perceived level of stress (Table 4). The odds of being assessed with upper quartile for stress was 1.4 times (95% CI 1.02–1.86, *p* = 0.037) higher in the high-risk group compared with the low-risk group. Furthermore, the proportion of staff in the high-risk group with upper quartile stress was higher than that in the low-risk group (24.7% *v*. 19.3%, *p* = 0.037). The average level of perceived stress status was also significantly higher in the high-risk group [PSS: 27 (20–29) *v.* 25.5 (19–28.75)], with a difference of 1.5 (*p* = 0.015) between groups.

**Table 4.** Variables associated with the hospital staff's perceived stress level (*N* = 1045)

Independent variables	PSS ⩽ 29^a^ (*N* = 822)	PSS > 29 (*N* = 223)	Univariate analyses	
			Crude OR (95% CI)^b^	*p*-value
Gender, *n* (%)				
Male	123 (83.1)	25 (16.9)	0.72 (0.45–1.13)	0.15
Female	699 (77.9)	198 (22.1)	–	–
Education level, *n* (%)				
Junior college	311 (75.7)	100 (24.3)	–	–
College	474 (80.5)	115 (19.5)	0.76 (0.56–1.02)	0.07
Master and above	37 (82.2)	8 (17.8)	0.67 (0.30–1.49)	0.33
Job, *n* (%)				
Doctor	113 (75.8)	36 (24.2)	1.74 (0.94–3.23)	0.08
Nurse	605 (78.3)	168 (21.7)	1.52 (0.91–2.55)	0.11
Others	104 (84.6)	19 (15.4)	–	–
Years of working, *n* (%)				
<1 year	64 (79.0)	17 (21.0)	1.03 (0.59–1.82)	0.92
2–3 years	123 (76.9)	37 (23.1)	1.17 (0.77–1.76)	0.46
4–5 years	92 (76.0)	29 (24.0)	1.22 (0.77–1.93)	0.39
>5 years	543 (79.5)	140 (20.5)	–	–
Department (multiple choices), *n* (%)				
Fever clinic	54 (71.1)	22 (28.9)	0.64 (0.38–1.08)	0.09
Emergency	94 (76.4)	29 (23.6)	0.86 (0.55–1.35)	0.52
ICU	34 (75.6)	11 (24.4)	0.83 (0.41–1.67)	0.60
Infections department	61 (78.2)	17 (21.8)	0.97 (0.56–1.70)	0.92
Others	616 (79.8)	156 (20.2)	1.28 (0.93–1.78)	0.13
Work environment, *n* (%)				
High risk	302 (75.3)	99 (24.7)	1.38 (1.02–1.86)	0.037
Low risk	520 (80.7)	124 (19.3)	–	–

PSS, Perceived Stress Scale; ICU, intensive care unit.

a As reference.

b Odds ratios (95% CI).

### Factors associated with medical staff's sleep status

Nearly half (49.9%) of the respondents reported insomnia symptoms (ISI ⩾ 8). In total, 109 (10.4%) respondents had a clinical sleep disorders and presented with ISI scores ⩾15. The proportion of staff in the high-risk group with insomnia symptoms (56.6%) and clinical insomnia (13.5%) was higher than that of the low-risk group [45.8% (*p* = 0.001) and 8.5% (*p* = 0.011), respectively]. Median ISI scores were also higher [9 (6–13) *v.* 7 (4–12), *p* < 0.001]. The odds of being assessed with a higher degree of insomnia was 1.60 times (95% CI 1.07–2.40, *p* = 0.023) greater in the high-risk group than the low-risk group. The odds of being assessed with a higher degree of insomnia were 1.88 times (95% CI 1.09–3.26, *p* = 0.023) greater among staff with fewer years of employment in the current role, compared to those with a longer employment. Staff with a higher education level also had a lower chance of suffering from insomnia (*p* = 0.027; Table 5).

**Table 5.** Variables associated with the hospital staff's sleep status (*N* = 1045)

Independent variables	ISI ⩽ 14^a^ (*N* = 936)	ISI > 14 (*N* = 109)	Univariate analyses		Logistic regression analysis^b^	
			Crude OR (95% CI)^b^	*p*-value	Adjusted OR (95% CI)^c^	*p*-value
Gender, *n* (%)						
Male	133 (89.9)	15 (10.1)	0.96 (0.54–1.71)	0.90	0.94 (0.51–1.74)	0.85
Female	803 (89.5)	94 (10.5)	–	–	–	–
Education level, *n* (%)						
Junior college	357 (86.9)	54 (13.1)	–	–	–	–
College	541 (91.9)	48 (8.1)	0.59 (0.39–0.89)	0.011	0.61 (0.39–0.94)	0.027
Master and above	38 (84.4)	7 (15.6)	1.22 (0.52–2.87)	0.65		
Job, *n* (%)						
Doctor	132 (88.6)	17 (11.4)	2.13 (0.86–5.33)	0.10		
Nurse	688 (89.0)	85 (11.0)	2.05 (0.92–4.54)	0.08		
Others	116 (94.3)	7 (5.7)	–	–		
Years of working, *n* (%)						
<1 year	74 (91.4)	7 (8.6)	0.95 (0.42–2.15)	0.90		
2–3 years	141 (88.1)	19 (11.9)	1.35 (0.78–2.33)	0.28		
4–5 years	100 (82.6)	21 (17.4)	2.10 (1.23–3.60)	0.007	1.88 (1.09–3.26)	0.023
>5 years	621 (90.9)	62 (9.1)	–	–	–	–
Department (multiple choices), *n* (%)						
Fever clinic	66 (86.8)	10 (13.2)	1.33 (0.66–2.67)	0.42		
Emergency	104 (84.6)	19 (15.4)	1.69 (0.99–2.88)	0.06		
ICU	39 (86.7)	6 (13.3)	1.34 (0.55–3.24)	0.52		
Infections department	73 (93.6)	5 (6.4)	0.57 (0.23–1.44)	0.23		
Others	697 (90.3)	75 (9.7)	0.76 (0.49–1.16)	0.20		
Work environment, *n* (%)						
High risk	347 (86.5)	54 (13.5)	1.67 (1.12–2.48)	0.012	1.60 (1.07–2.40)	0.023
Low risk	589 (91.5)	55 (8.5)	–	–	–	–

ISI, Insomnia Severity Index; ICU, intensive care unit.

a As reference.

b Multivariate logistic regression analysis with dependent variable and independent variables ‘gender and the statistically significant variables of the univariate comparisons’.

c Odds ratios (95% CI).

### The relationships between the HADS, PSS, and ISI of the medical staff

There was a significant and positive correlation between HADS-A and HAS-D scores (*r*_s_ = 0.69, *p* < 0.001), HADS-A and PSS scores (*r*_s_ = 0.63, *p* < 0.001), and HADS-A and ISI scores (*r*_s_ = 0.48, *p* < 0.001). There was also a significant and positive correlation between the HADS-D and PSS scores (*r*_s_ = 0.65, *p* < 0.001), HADS-D and the ISI scores (*r*_s_ = 0.42, *p* < 0.001). And there was a significant and positive correlation between PSS and ISI scores (*r*_s_ = 0.44, *p* < 0.001).

## Discussion

The objective of this study was to report on the psychological impact of the COVID-19 pandemic on medical staff in the Guangdong province of China using a cross-sectional survey conducted at four large hospitals. To our knowledge, this is one of the earliest evaluations of psychological distress in medical staff treating COVID-19 patients (Huang & Zhao, 2020; Lai et al., 2020; Tang et al., 2020; Xiao, Zhang, Kong, Li, & Yang, 2020). The results of the current study show that in early February 2020, when there were significant public and medical staff concerns about a novel infectious disease outbreak, 40–50% of the surveyed medical staff had anxiety or depressive symptoms, and nearly half reported insomnia symptoms.

A significant proportion (38.4%) of medical staff had already been in contact with ⩾1 confirmed or suspected case of COVID-19 at the time of the survey, and this group did not only comprise staff from the anticipated ‘front-line’ departments. Staff classified as having a lower risk of infection reported a lower average level of anxiety, depression, perceived stress status, and insomnia severity than those in the high-risk group; however, they had a similar incidence of severe anxiety. Interestingly, doctors were particularly susceptible to moderate-severe depression. Scores for anxiety (HADS-A), depression (HADS-D), stress (PSS), and insomnia (ISI) were all significantly correlated.

Medical staff are known to experience significant stress during infectious epidemics (Lehmann et al., 2016; Lin & Li, 2020; Matsuishi et al., 2012; Maunder, 2004; Wu et al., 2009). Reports of the psychological impact of SARS indicated that high levels of distress were common among medical staff in Toronto (Maunder et al., 2004). Our current study found a significant percentage of medical staff suffered from mood disorder symptoms, including anxiety and depression (47.8% and 39.4%), during the COVID-19 influenza pandemic in Guangdong province, mirroring that seen in previous studies conducted in the Fujian province and the regions outside Hubei province (35–40%) (Huang & Zhao, 2020; Lai et al., 2020). However, it seems that the prevalence of anxiety and depression among medical staff in Wuhan (the epicentre of the current outbreak) was even higher (about 50–60%) (Lai et al., 2020). Together, these findings suggest that mental health burden among medical staff during the COVID-19 pandemic is highly correlated with the severity of the pandemic at their workplace.

Close person-to-person contact seems to be the primary mode of transmission for COVID-19 (Li et al., 2020). It is believed COVID-19 is transmitted most readily by respiratory droplets, but the virus also can spread when people touch a surface or object contaminated with infectious droplets and then touch their mouth, nose, or eyes (Lin & Li, 2020). During our study, doctors and nurses were being equipped with full-body protective gear to be exchanged every 4–6 h. To avoid becoming infected when removing the protective equipment, the staff could not eat, drink, or use the bathroom during their working hours. Combining these working conditions with the requirement of staff in isolation wards to have frequent and close patient contact, it is easy to envisage that staff could easily grow mentally and physically exhausted. We suggest that this type of working environment may predispose staff to anxious and depressive emotional disturbances, as well as somatic symptoms.

Nearly half of the respondents in our survey self-reported insomnia, and the perceived stress status and insomnia severity was higher in staff in the high-risk grouping (who had potential or direct contact with infectious patients). A similar situation has previously been noted in research by Chen et al. (2006), and Maunder, Hunter, Vincent, Bennett, and Mazzulli (2003), where medical staff exhibited poor sleep quality during the SARS outbreak. We found that staff with more experience in their role had, on average, lower levels of insomnia (negative correlation); possibly because they were more accustomed to the relevant occupational hazards and had been able to develop resilience to high-stress and high-intensity working conditions. A previous study similarly demonstrated that nurses with less experience are more likely to experience sleeplessness during a highly infectious work situation than those with more experience (Khatony, Zakiei, Khazaie, Rezaei, & Janatolmakan, 2020).

When faced with a pandemic, not only the conventional front-line staff (in departments such as the fever clinic, emergency department, ICU, or infections disease department) might have a high risk of exposure – others might do too. In our study, around a quarter of the high-risk grouping (which was based on contact) consisted of staff working in other departments. We found that staff in the high-risk group had a similar chance of having severe anxiety than those in the low-risk group. These results are similar to those of Chen, Wu, Yang, and Yen (2005) in Taiwan, China. Additionally, anxiety and depression dimensions measuring by General Severity Index during the Ebola outbreak showed no differences between high and low exposures risk staff (Ji, Ji, Duan, Li, & Duan, 2017). There are several possible explanations for this result. Firstly, exposure to high-intensity and high-risk work settings (such as ICUs and emergency department work), and direct exposure to infected patients, have been shown not to be the primary determinants of adverse psychological outcomes (Maunder et al., 2003); rather, the resilience of medical staff who choose this type of work was. Secondly, a high level of personal protection training, professional experience, and good social support both before and during the outbreak might have buffered the average severity of symptoms among staff in the high-risk group (Dyregrov, Kristoffersen, & Gjestad, 1996; Weisæth, 2007). Additionally, while the fear around the potential infection (and consequences) applies to all staff, and the actual risk might be higher in those with increased exposure, staff in the low-risk group may also have concerns about understaffing/overworking etc., and their lack of active control around the situation. The interpersonal isolation and stigmatisation from the society might make it worse. A few news reports had reported that some communities claim to prevent medical staff from going home for safety reason.

Our findings show a positive correlation between the level of anxiety and depression with both insomnia, and stress perceived level, among medical staff working during the recent COVID-19 pandemic. This is similar to the previous study of Xiao et al. (2020). There is growing evidence that sleep problems are observed in mood disorders such as depression and anxiety (Cox & Olatunji, 2020; Ford & Kamerow, 1989), and that the persistence of stress or trauma can lead to the development of stress-induced illnesses such as depression and anxiety (Kendler et al., 2000). These findings may relate to chronic sensitivity of the hypothalamic–pituitary–adrenocortical axis and chronic over-activation of the sympathetic–adrenal–medulla system; the two main biological mechanisms involved in the reaction to stress and also the pathophysiology of sleep disturbances (Meerlo, Sgoifo, & Suchecki, 2008). It has been suggested that social support (such as a wide social network) could reduce anxiety and stress, improve self-efficacy, and indirectly improve sleep quality (Xiao et al., 2020). These findings may provide some ideas for ways to prevent and reduce the psychological impact of infectious outbreaks in the future.

The main methodological limitation of the current study was potential sampling bias that may have overestimated the prevalence of psychological symptoms. This is because respondents in mental distress may have been more interested and willing to complete the questionnaire, and the cover note explaining the purpose of the survey may have skewed participant responses and study results. The second potential limitation is that our findings might not be generalisable to other healthcare settings or time periods within the current COVID-19 pandemic. This study represents a snapshot of the situation in the specified regions representing developed area, sub-developed area and less developed area respectively in Guangdong province. Further longitudinal studies in other geographical regions and in different healthcare systems are needed.

## Conclusion

An efficient public health depends on the physical and mental well-being of its medical staff. We found that a significant proportion of medical staff responding to COVID-19 outbreak in Guangdong Province, China, experienced moderate-severe levels of anxiety and depression during a snapshot in time from early in the pandemic. Perceived stress status and insomnia severity was higher in staff with more risk of infection than those with a less risk, but the prevalence of severe anxiety was similar. We suggest that medical protective equipment and psychological stress assistance should be offered to all medical staff during infectious disease outbreaks, including those indirectly involved. As previously suggested by Chen et al. (2006), a systematic prevention programme that included a series of in-service training, detailed manpower allocation, adequate protective equipment, and the availability of a mental health team is feasible and effective. The current COVID-19 pandemic is unlikely to be the last outbreak of a highly infectious disease and it is important to prepare our health care systems for the future.

## Supplementary material

For supplementary material accompanying this paper visit http://dx.doi.org/10.1017/S0033291720002561. click here to view supplementary material
