# Supplementary material for: The psychological impact of COVID-19 pandemic on medical staff in Guangdong, China: a cross-sectional study
Source: Psychol Med. 2020 Jul 6:1–9. doi: 10.1017/S0033291720002561 (PMC7371926; doi:10.1017/S0033291720002561)
Supplement: Supplementary file 1 [file S0033291720002561sup.zip › S0033291720002561sup001.docx]

**Additional File 1 ：**

(English Version)

**Hospital Anxiety and Depression Scale (HADS)**

**Introduction：** Tick the box beside the reply that is closest to how you have been feeling in the past 2 week. Don’t take too long over you replies:

| **D** | **A** |  | **D** | **A** |  |
| --- | --- | --- | --- | --- | --- |
|  |  | **I feel tense or 'wound up':** |  |  | **I feel as if I am slowed down:** |
|  | 3 | Most of the time | 3 |  | Nearly all the time |
|  | 2 | A lot of the time | 2 |  | Very often |
|  | 1 | From time to time, occasionally | 1 |  | Sometimes |
|  | 0 | Not at all | 0 |  | Not at all |
|  |  |  |  |  |  |
|  |  | **I still enjoy the things I used to enjoy:** |  |  | **I get a sort of frightened feeling like 'butterflies' in the stomach:** |
| 0 |  | Definitely as much |  | 0 | Not at all |
| 1 |  | Not quite so much |  | 1 | Occasionally |
| 2 |  | Only a little |  | 2 | Quite Often |
| 3 |  | Hardly at all |  | 3 | Very Often |
|  |  |  |  |  |  |
|  |  | **I get a sort of frightened feeling as if something awful is about to**  **happen:** |  |  | **I have lost interest in my appearance:** |
|  | 3 | Very definitely and quite badly | 3 |  | Definitely |
|  | 2 | Yes, but not too badly | 2 |  | I don't take as much care as I should |
|  | 1 | A little, but it doesn't worry me | 1 |  | I may not take quite as much care |
|  | 0 | Not at all | 0 |  | I take just as much care as ever |
|  |  |  |  |  |  |
|  |  | **I can laugh and see the funny side of things:** |  |  | **I feel restless as I have to be on the**  **move:** |
| 0 |  | As much as I always could |  | 3 | Very much indeed |
| 1 |  | Not quite so much now |  | 2 | Quite a lot |
| 2 |  | Definitely not so much now |  | 1 | Not very much |
| 3 |  | Not at all |  | 0 | Not at all |
|  |  |  |  |  |  |
|  |  | **Worrying thoughts go through my mind:** |  |  | **I look forward with enjoyment to**  **things:** |
|  | 3 | A great deal of the time | 0 |  | As much as I ever did |
|  | 2 | A lot of the time | 1 |  | Rather less than I used to |
|  | 1 | From time to time, but not too often | 2 |  | Definitely less than I used to |
|  | 0 | Only occasionally | 3 |  | Hardly at all |
|  |  |  |  |  |  |
|  |  | **I feel cheerful:** |  |  | **I get sudden feelings of panic:** |
| 3 |  | Not at all |  | 3 | Very often indeed |
| 2 |  | Not often |  | 2 | Quite often |
| 1 |  | Sometimes |  | 1 | Not very often |
| 0 |  | Most of the time |  | 0 | Not at all |
|  |  |  |  |  |  |
|  |  | **I can sit at ease and feel relaxed:** |  |  | **I can enjoy a good book or radio or TV program:** |
|  | 0 | Definitely | 0 |  | Often |
|  | 1 | Usually | 1 |  | Sometimes |
|  | 2 | Not Often | 2 |  | Not often |
|  | 3 | Not at all | 3 |  | Very seldom |

(Chinese Version)

**医院焦虑抑郁量表**

**指导语：**请您根据最近一个月的实际情况填写以下问题。

| D | A |  | D | A |  |
| --- | --- | --- | --- | --- | --- |
|  |  | 1.我感到紧张(或痛苦) |  |  | 8.我对自己的仪容(打扮自己)失去兴趣： |
|  | 3 | 几乎所有时候 | 3 |  | 肯定 |
|  | 2 | 大多数时候 | 2 |  | 并不象我应该做到的那样关心 |
|  | 1 | 有时 | 1 |  | 我可能不是非常关心 |
|  | 0 | 根本没有 | 0 |  | 我仍象以往一样关心 |
|  |  |  |  |  |  |
|  |  | 2.我对以往感兴趣的事情还是有兴趣： |  |  | 9.我有点坐立不安，好象感到非要活动不可： |
| 0 |  | 肯定一样 |  | 3 | 确实非常多 |
| 1 |  | 不像以前那样多 |  | 2 | 是不少 |
| 2 |  | 只有一点儿 |  | 1 | 并不很多 |
| 3 |  | 基本上没有了 |  | 0 | 根本没有 |
|  |  |  |  |  |  |
|  |  | 3.我感到有点害怕，好象预感到有什么可怕事情要发生： |  |  | 10.我对一切都是乐观地向前看： |
|  | 3 | 非常肯定和十分严重 | 0 |  | 差不多是这样做的 |
|  | 2 | 是有，但并不太严重 | 1 |  | 并不完全是这样做的 |
|  | 1 | 有一点，但并不使我苦恼 | 2 |  | 很少这样做 |
|  | 0 | 根本没有 | 3 |  | 几乎从来不这样做 |
|  |  |  |  |  |  |
|  |  | 4.我能够哈哈大笑，并看到事物好的一面： |  |  | 11.我突然发现恐慌感： |
| 0 |  | 我经常这样 |  | 3 | 确实很经常 |
| 1 |  | 现在已经不大这样了 |  | 2 | 时常 |
| 2 |  | 现在肯定是不太多了 |  | 1 | 时常 |
| 3 |  | 根本没有 |  | 0 | 根本没有 |
|  |  |  |  |  |  |
|  |  | 5.我的心中充满烦恼： |  |  | 12.我好象感到情绪在渐渐低落： |
|  | 3 | 大多数时间 | 3 |  | 几乎所有的时间 |
|  | 2 | 常常如此 | 2 |  | 很经常 |
|  | 1 | 时时，但并不经常 | 1 |  | 有时 |
|  | 0 | 偶然如此 | 0 |  | 根本没有 |
|  |  |  |  |  |  |
|  |  | 6.我感到愉快： |  |  | 13.我感到有点害怕，好象某个内脏器官变坏了： |
| 3 |  | 根本没有 |  | 0 | 根本没有 |
| 2 |  | 并不经常 |  | 1 | 有时 |
| 1 |  | 有时 |  | 2 | 很经常 |
| 0 |  | 大多数 |  | 3 | 非常经常 |
|  |  |  |  |  |  |
|  |  | 7.我能够安闲而轻松地坐着： |  |  | 14.我能欣赏一本好书或一项好的广播或电视节目： |
|  | 0 | 肯定 | 0 |  | 常常 |
|  | 1 | 经常 | 1 |  | 有时 |
|  | 2 | 并不经常 | 2 |  | 并非经常 |
|  | 3 | 根本没有 | 3 |  | 很少 |
