# Supplementary material for: The psychological impact of COVID-19 pandemic on medical staff in Guangdong, China: a cross-sectional study
Source: Psychol Med. 2020 Jul 6:1–9. doi: 10.1017/S0033291720002561 (PMC7371926; doi:10.1017/S0033291720002561)
Supplement: Supplementary file 1 [file S0033291720002561sup.zip › S0033291720002561sup002.docx]

**Additional File 2 ：**

(English Version)

**PERCEIVED STRESS SCALE (PSS-14)**

**Instruction：**The questions in this scale ask you about your feelings and thoughts during the last month. In each case, you will be asked to indicate by circling how often you felt or thought a certain way.

| **0 = Never 1 = Almost Never 2 = Sometimes 3 = Fairly Often 4 = Very Often** | | | | | |
| --- | --- | --- | --- | --- | --- |
| 1. In the last month, how often have you been upset because of something that happened unexpectedly? | 0 | 1 | 2 | 3 | 4 |
| 2. In the last month, how often have you felt that you were unable to control the important things in your life? | 0 | 1 | 2 | 3 | 4 |
| 3. In the last month, how often have you felt nervous and “stressed”? | 0 | 1 | 2 | 3 | 4 |
| 4. In the last month, how often have you successfully dealt with the hassles of life? | 0 | 1 | 2 | 3 | 4 |
| 5. In the last month, how often have you felt that you are effectively dealing with the important changes in your life? | 0 | 1 | 2 | 3 | 4 |
| 6. In the last month, how often have you felt confident about your ability to handle your personal problems? | 0 | 1 | 2 | 3 | 4 |
| 7. In the last month, how often have you felt that things were going your way? | 0 | 1 | 2 | 3 | 4 |
| 8. In the last month, how often have you found that you could not cope with all the things that you had to do? | 0 | 1 | 2 | 3 | 4 |
| 9. In the last month, how often have you been able to control irritations in your life? | 0 | 1 | 2 | 3 | 4 |
| 10. In the last month, how often have you felt that you were on top of things? | 0 | 1 | 2 | 3 | 4 |
| 11. In the last month, how often have you been angered because of things that were outside of your control? | 0 | 1 | 2 | 3 | 4 |
| 12. In the last month, how often have you thought of the things you have to do? | 0 | 1 | 2 | 3 | 4 |
| 13. In the last month, how often have you take good command of time management? | 0 | 1 | 2 | 3 | 4 |
| 14. In the last month, how often have you felt difficulties were piling up so high that you could not overcome them? | 0 | 1 | 2 | 3 | 4 |

| **0 = 从不 1 = 偶尔 2 = 有时 3 = 时常 4 = 总是** | | | | | |
| --- | --- | --- | --- | --- | --- |
| 1.一些无法预期的事情发生而感到心烦意乱 | 0 | 1 | 2 | 3 | 4 |
| 2.感觉无法控制自己生活中重要的事情 | 0 | 1 | 2 | 3 | 4 |
| 3.感到紧张不安和压力 | 0 | 1 | 2 | 3 | 4 |
| 4.成功地处理恼人的生活麻烦 | 0 | 1 | 2 | 3 | 4 |
| 5.感到自己是有效地处理生活中所发生的重要改变 | 0 | 1 | 2 | 3 | 4 |
| 6.对于有能力处理自己私人的问题感到很有信心 | 0 | 1 | 2 | 3 | 4 |
| 7.感到事情顺心如意 | 0 | 1 | 2 | 3 | 4 |
| 8.发现自己无法处理所有自己必须做的事情 | 0 | 1 | 2 | 3 | 4 |
| 9.有办法控制生活中恼人的事情 | 0 | 1 | 2 | 3 | 4 |
| 10.常觉得自己是驾驭事情的主人 | 0 | 1 | 2 | 3 | 4 |
| 11.常生气，因为很多事情的发生是超出自己所能控制的 | 0 | 1 | 2 | 3 | 4 |
| 12.经常想到有些事情是自己必须完成的 | 0 | 1 | 2 | 3 | 4 |
| 13.常能掌握时间安排方式 | 0 | 1 | 2 | 3 | 4 |
| 14.常感到困难的事情堆积如山，而自己无法克服它们 | 0 | 1 | 2 | 3 | 4 |

(Chinese Version)

**压力感知量表-14条目**

**指导语：**请您根据最近一个月的实际情况填写以下问题。
