# Supplementary material for: The psychological impact of COVID-19 pandemic on medical staff in Guangdong, China: a cross-sectional study
Source: Psychol Med. 2020 Jul 6:1–9. doi: 10.1017/S0033291720002561 (PMC7371926; doi:10.1017/S0033291720002561)
Supplement: Supplementary file 1 [file S0033291720002561sup.zip › S0033291720002561sup003.docx]

**Additional File 3 ：**

(English Version)

**Insomnia Severity Index (ISI)**

**Instruction：**The questions in this scale ask you about your feelings and thoughts during the last 2 week. In each case, you will be asked to indicate by circling how often you felt or thought a certain way.

| 1. The severity of your current insomnia problem |  |  |  |  |  |
| --- | --- | --- | --- | --- | --- |
| a.Difficulty in falling asleep | none | mild | moderate | severe | very severe |
| b.Difficulty in the maintenance of their sleep | none | mild | moderate | severe | very severe |
| c.Problem waking up too early | none | mild | moderate | severe | very severe |
| 2. How satisfied or dissatisfied were you with your present sleep pattern | very satisfied | satisfied | moderately satisfied | dissatisfied | very dissatisfied |
| 1. How was the impairment in quality of your life owing to insomnia | not at all | a little | somewhat | much | very much |
| 1. How worried/distressed was you about your current sleep problems | not at all worried | a little | somewhat | much | very much worried |
| 1. How adversely do your daily functioning (e.g., daytime fatigue, mood, ability to function at work/daily chores, concentration, memory, and mood) has been interfered with by your current sleep problems. | not at all interfering | a little | somewhat | much | very much |

(Chinese Version)

**失眠严重指数量表**

**指导语：**请您根据最近2周的实际情况填写以下问题。

| 1.描述你当前失眠问题的严重程度 |  |  |  |  |  |
| --- | --- | --- | --- | --- | --- |
| a.入睡困难 | 无 | 轻度 | 中度 | 重度 | 极重度 |
| b.维持睡眠困难 | 无 | 轻度 | 中度 | 重度 | 极重度 |
| c.早醒 | 无 | 轻度 | 中度 | 重度 | 极重度 |
| 2.对你当前睡眠模式的满意度 | 很满意 | 满意 | 一般 | 不满意 | 很不满意 |
| 3.你对自己当前睡眠问题有多大程度的焦虑和烦扰 | 没有 | 一点 | 有些 | 较多 | 很多 |
| 4.与其他人相比，你的失眠问题对你的生活质量有多大程度的影响或损害 | 没有 | 一点 | 有些 | 较多 | 很多 |
| 5.你认为你的睡眠问题在多大程度上干扰了你的日间功能（如日间疲劳、除了工作和日常事务的能力、注意力、记忆力、情绪等） | 没有干扰 | 轻微干扰 | 有些干扰 | 较多干扰 | 很多干扰 |
